# Pesticide Exposure as a Risk Factor for Myelodysplastic Syndromes: A Meta-Analysis Based on 1,942 Cases and 5,359 Controls

**DOI:** 10.1371/journal.pone.0110850

**Published:** 2014-10-21

**Authors:** Jie Jin, Mengxia Yu, Chao Hu, Li Ye, Lili Xie, Jin Jin, Feifei Chen, Hongyan Tong

**Affiliations:** 1 Department of Hematology, the First Affiliated Hospital of Zhejiang University, Hangzhou, People’s Republic of China; 2 Institute of Hematology, Zhejiang University School of Medicine, Hangzhou, People's Republic of China; 3 Myelodysplastic syndromes diagnosis and therapy center, Zhejiang University School of Medicine, Hangzhou, People's Republic of China; Carleton University, Canada

## Abstract

**Objective:**

Pesticide exposure has been linked to increased risk of cancer at several sites, but its association with risk of myelodysplastic syndromes (MDS) is still unclear. A meta-analysis of studies published through April, 2014 was performed to investigate the association of pesticide exposure with the risk of MDS.

**Methods:**

Studies were identified by searching the Web of Science, Cochrane Library and PubMed databases. Summary odds ratios (ORs) with corresponding 95% confidence intervals (CIs) were calculated using random- or fixed-effect models.

**Results:**

This meta-analysis included 11 case-control studies, all of which demonstrated a correlation between pesticide exposure and a statistically significant increased risk of MDS (OR = 1.95, 95% CI 1.23–3.09). In subgroup analyses, patients with pesticide exposure had increased risk of developing MDS if they were living in the Europe or Asia and had refractory anemia (RA) or RA with ringed sideroblasts (RARS). Moreover, in the analysis by specific pesticides, increased risk was associated with exposure to insecticides (OR = 1.71, 95% CI 1.22–2.40) but not exposure to herbicides or fungicides.

**Conclusion:**

This meta-analysis supports the hypothesis that exposure to pesticides increases the risk of developing MDS. Further prospective cohort studies are warranted to verify the association and guide clinical practice in MDS prevention.

## Introduction

Myelodysplastic syndromes (MDS) are a heterogeneous group of stem cell malignancies, characterized by ineffective hematopoiesis, and peripheral blood cytopenias. With disease progression, the risk of transformation into acute myeloid leukemia (AML) increased [1], [2]. Despite development of new therapeutic methods in recent years, treatment of MDS is still limited and MDS remains incurable except in the case of the younger patients with good performance status, allogeneic stem cell transplantation eligibility, and adequate donor access [3]. As the whole population ages, MDS will become one of the most common myeloid malignancies. The societal impact and burden of the disease, measured in terms of the number of people affected yearly with a new diagnosis or who are living with the disease, is enormous and will continue to increase in the future. Therefore, a better comprehension of the etiology and further investigation of risk factors may significantly improve MDS prevention measures and reduce MDS incidence.

Since 1950, pesticide use has risen over 50% and pesticide toxicity has increased ten-fold [4]. Pesticide exposure is thought to increase cancer risk by promoting oxidative stress, chromosomal aberrations, cell signaling disturbances or gene mutations [5], [6], [7].

Over the past few decades, some epidemiological studies have analyzed the association between pesticide exposure and risk of MDS, but the findings are controversial. Five studies showed a positive association between incidence of MDS and pesticide exposure [8], [9], [10], [11], [12], and six studies illustrated no association [13], [14], [15], [16], [17], [18]. Hence, the present meta-analysis was undertaken to further examine the potential involvement of pesticide exposure in MDS etiology.

## Materials and Methods

### Literature research

A systematic literature search of the Web of Science, Cochrane Library and PubMed was executed by two independent reviewers (Chao Hu and Mengxia Yu). The following search strategy was used: (myelodysplastic syndrome OR MDS OR myelodysplastic OR myelodysplasia OR preleukemia) AND (pesticides OR herbicides OR fungicides OR insecticides). All relevant titles or abstracts were screened (see Study selection) to determine the suitability of each publication, and full-text articles were retrieved. We also checked the references from retrieved articles for additional studies not identified by database search.

### Study selection

Studies included in this meta-analysis had to meet all the following criteria: (a) one of the exposures of interest was pesticide exposure; (b) one of the outcomes of interest was incidence of MDS; (c) a cohort design or case-control design; (d) providing the risk and corresponding 95% confidence intervals (CIs) or data to calculate these; (e) written in the English language. If there were multiple publications from the same study or overlapping study populations, the most recent and detailed study was eligible for inclusion in the meta-analysis.

### Data extraction

Data were collected independently by two reviewers using a predefined data collection form. The following data were extracted from each study and included in the final analysis: the study name (together with the first author’s name and year of publication), country of origin, gender, age, study design, source of patients, number of cases/controls, risk factor assessment, matching covariates, and adjusted covariates. We contacted the corresponding authors of the primary studies to acquire missing or insufficient data (when necessary), used group consensus and consulted a third reviewer to resolve discrepancies, and assigned scores of <7 and ≥7 for low- and high-quality studies, respectively, on the nine-score Newcastle-Ottawa Scale (NOS) [19], [20].

### Statistical analysis

To determine whether to use the fixed- or random-effects model, we measured statistical heterogeneity [21]. A fixed-effects model was used to calculate a pooled odds ratio (OR) with 95% CI when there was no heterogeneity. Otherwise, we calculated pooled ORs and confidence intervals assuming a random-effects model. The homogeneity of ORs across individual studies was quantified by the Q statistic and the *I^2^* score. *P*>0.05 for the Q-test was considered as a lack of heterogeneity among the studies. The *I^2^* values of 25%, 50%, and 75% represented mild, moderate, and severe heterogeneity, respectively [22]. Potential publication bias was assessed by using Begg’s funnel plots (rank correlation method where an asymmetrical plot suggested possible publication bias) [23] and Egger’s bias test (linear regression method where *P*<0.05 indicated the presence of statistically significant publication bias) [24]. Sensitivity analysis was conducted, in which the meta-analysis estimates were calculated by sequential omission of every study in turn, so as to reflect the influence of the data from individual studies on the pooled ORs and evaluate the stability of the results. Cumulative meta-analysis was also conducted by sorting the studies based on publication time. Subset analyses were performed by source of patients, disease subtype, geographic region, study quality, and type of pesticide. All of the statistical analyses were performed with STATA 11.0 (Stata Corporation, College Station, TX) using two-sided *P*-values, where *P*<0.05 was considered statistically significant.

## Results

### Literature search and study characteristics

**Figure 1:**
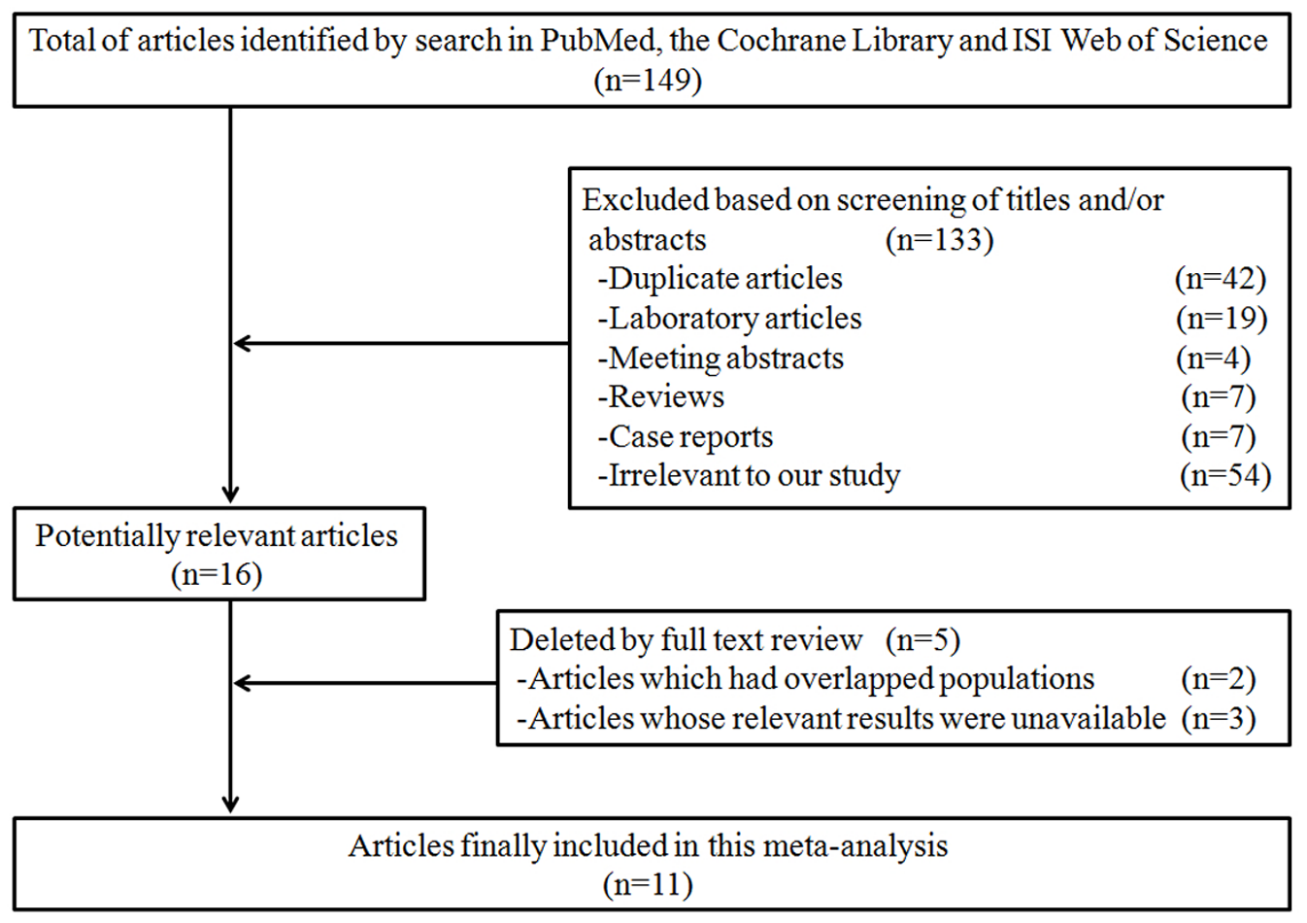
Process of study selection.

The results of our literature search strategy and study selection process were detailed in Figure 1. We identified 11 case-control studies on the association of pesticide exposure with risk of MDS published between 1990 and 2011 [8], [9], [10], [11], [12], [13], [14], [15], [16], [17], [18]. A total of 1,942 MDS patients and 5,359 controls were included in the present meta-analysis. Among the 5,359 controls, 3853 persons were hospitalized patients without conditions related to hematological diseases and the remaining 1506 were recruited from healthy people. A total of 1456 participants have exposed to pesticide, of whom 323 suffered from MDS. Among the chosen studies, seven were conducted in Europe, three in United States, and one in Asia. Pesticide exposure was ascertained by interview or questionnaire or both. The study quality was graded by the Newcastle-Ottawa Quality Assessment Scale, ranged from 5 to 8 (with a mean of 6). The main characteristics of the included articles were listed in Table 1.

**Table 1:** Main characteristics of studies evaluating the association between pesticides exposure and MDS.

Study	Country	Gender	Age	StudyDesign	Sourceof patients	Numberof cases	Numberof controls	Risk factorAssessment	StudyQuality	Matching and Adjustments
Kokouva(2011)^13^	Greece	M/F	27–73	Case-control	Hospital-based	78	455	Questionnaire	5	Gender, age, smoking, family history
Lv(2011)^14^	China	M/F	20–88	Case-control	Hospital-based	403	806	Face-to-faceInterview	6	Age, sex, anti-tb drugs, D860, traditional Chinesemedicine, alcohol intake, benzene, gasoline,glues, hair dye, education, new building
Pekmezovic(2006)^8^	SerbiaMontenegro	M/F	18–85	Case-control	Hospital-based	80	160	Interview	6	Age, sex
Strom(2005)^9^	United States	M/F	24–89	Case-control	Hospital-based	354	452	Mailedquestionnaire	7	Age, sex, ethnicity, education, family history ofhematopoietic cancer, alcohol intake, benzene,solvent, gasoline
Nisse(2001)^10^	France	M/F	NR	Case-control	Population-based	204	204	Interview	8	Agricultural workers, textile operators, healthprofessionals, living next to an industrial plant,commercial and technical sales representatives,machine operators, oil use, smoking
Rigolin(1998)^11^	Italy	M/F	17–85	Case-control	Hospital-based	178	178	Interview andquestionnaire	5	Age, sex
West(1995)^15^	UK	M/F	≥15	Case-control	Hospital-based	400	400	Interview andquestionnaire	6	Age, sex, area of residence and hospital,year of diagnosis
Mele(1994)^16^	Italy	M/F	≥15	Case-control	Hospital-based	111	1161	Interview	6	Age, sex, education, and residenceoutside study town
Ciccone(1993)^12^	Italy	M/F	15–74	Case-control	Hospital-based andpopulation-based	19	246	Interview	5	Sex, area of residence, age
Brown(1990)^17^	United States	M	≥30	Case-control	Population-based	63	1245	Interview	6	Vital status, age, state, tobacco use, family historyof lymphopoietic cancer, high-risk occupations andhigh-risk exposure
Goldberg(1990)^18^	United States	NR	28–88	Case-control	Hospital-based	52	52	Interview	6	Age and sex

M: male; F: female; NR: not reported; tb: tuberculosis.

### Risk estimation

**Figure 2:**
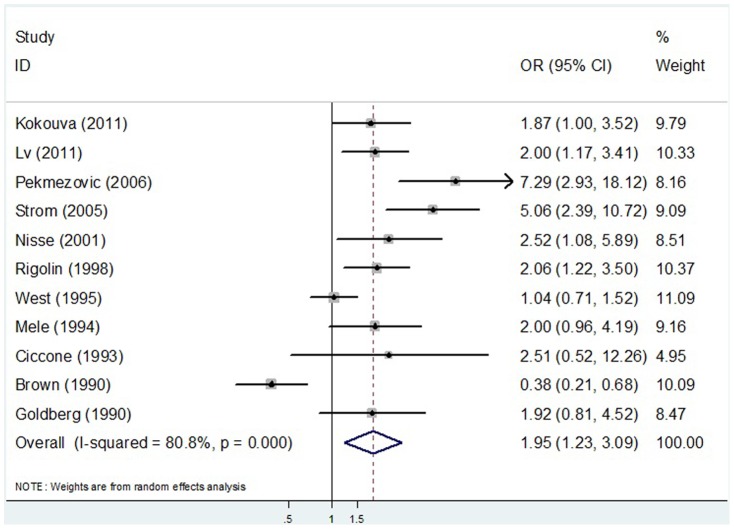
A forest plot illustrating risk estimates from included studies on the relationship between pesticide exposure and MDS risk.

**Figure 3:**
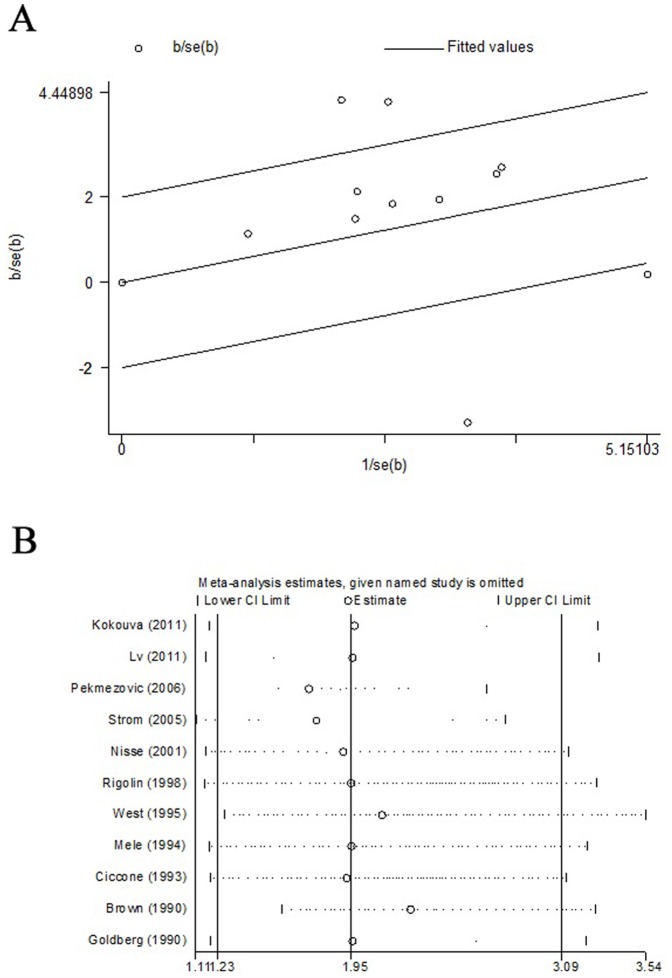
Evaluating the heterogeneity and the stability of the results. (A) Galbraith plot evaluating the heterogeneity; (B) Sensitivity analyses by sequential omission of individual studies in our study.

Our analysis demonstrated a significant adverse association between pesticide exposure (exposed vs. non-exposed status) and incidence of MDS (OR = 1.95, 95%CI 1.23–3.09) (Figure 2). Due to a statistically significant heterogeneity across studies (*I^2^* = 80.8%, *P*<0.001), the summary OR were estimated using the DerSimonian and Laird random effects model [25]. A Galbraith plot identified four studies as major sources of heterogeneity (Figure 3A). After excluding these four studies [8], [9], [15], [17], there was no study heterogeneity existed (*P* = 0.999, *I^2^* = 0.0%) and the overall association became stronger (OR = 2.04, 95% CI 1.57–2.66).

### Stratified analysis

Next, we pooled the OR estimates by patient source (population-based or hospital-based), MDS subtypes (refractory anemia (RA) and RA with ringed sideroblasts (RARS) or RA with excess blasts (RAEB) and RAEB in transformation (RAEBt)), geographic region (United States, Europe, or Asia), study quality (low or high), and type of pesticide (insecticide, herbicide or fungicide) (Table 2). When separated by patient source, the ORs (95% CI) were 2.26 (1.49–3.42) for hospital-based studies and 0.95 (0.15–6.06) for population-based studies. When stratified by MDS subtype, the associations were more positive for the RA/RARS sbutype (OR = 1.63, 95%CI 1.06–2.51) than the RAEB/RAEBt subtype (OR = 1.49, 95%CI 0.78–2.84). In the subset analyses stratified by geographic region, a statistically significant adverse effect of pesticide exposure on MDS was observed in Europe (OR = 2.13, 95%CI 1.35–3.36) and Asia (OR = 2.00, 95%CI 1.17–3.41), but not in United States (OR = 1.52, 95%CI 0.30–7.73). Furthermore, when stratified by study quality, the relationship was more significant in high quality studies (OR = 2.19, 95%CI 1.40–3.42) than in low quality studies (OR = 1.90, 95%CI 1.09–3.33). In addition, when analyzed by type of pesticide, the ORs (95% CI) for insecticides, herbicides, and fungicides were 1.71 (1.22–2.40), 1.16 (0.55–2.43) and 0.70 (0.20–3.20), respectively.

**Table 2:** Stratified pooled odds ratios of the relationship between pesticide exposure and risk of MDS.

Variables	Number of studies	Pooled OR(95%CI)	Q-test for heterogeneity*P* value (*I^2^* score)	Egger’s testP value	Begg’s testP value
Total	11 (8, 9, 10, 11, 12, 13, 14,15, 16, 17, 18)	1.95 (1.23–3.09)	<0.001 (80.8%)	0.350	0.113
Source of patients					
Population based	2 (10, 17)	0.95 (0.15–6.06)	<0.001 (92.3%)	–	1.000
Hospital based	8 (8, 9, 11, 13, 14, 15, 16, 18)	2.26 (1.49–3.42)	0.001 (71.7%)	0.098	0.266
Disease subtype					
RA/RARS	3 (9, 11, 18)	1.63 (1.06–2.51)	0.258 (25.6%)	0.413	0.734
RAEB/RAEBt	4 (9, 11, 14, 16)	1.49 (0.78–2.84)	0.005 (70.4%)	0.734	0.452
Geographic region					
Europe	7 (8, 10, 11, 12, 13, 15, 16)	2.13 (1.35–3.36)	0.006 (66.8%)	0.133	0.057
Asia	1 (14)	2.00 (1.17–3.41)	–	–	–
United States	3 (9, 17, 18)	1.52 (0.30–7.73)	<0.001 (93.4%)	0.407	1.000
Study quality					
High	2 (10, 11)	2.19 (1.40–3.42)	0.698 (0.0%)	–	1.000
Low	9 (8, 9, 12, 13, 14, 15, 16, 17, 18)	1.90 (1.09–3.33)	<0.001 (83.9%)	0.155	0.348
Type of pesticides					
Insecticides	9 (10, 11, 12, 13, 14, 15, 16, 17, 18)	1.71 (1.22–2.40)	0.009 (60.8%)	0.147	0.348
Herbicides	4 (14, 15, 16, 17)	1.16 (0.55–2.43)	0.056 (60.3%)	0.203	0.089
Fungicides	1 (17)	0.70 (0.20–3.20)	–	–	–

RA: refractory anemia; RARS: RA with ringed sideroblasts; RAEB: RA with excess blasts (RAEB); RAEBt: RAEB in transformation.

### Sensitivity analysis

We also carried out sensitivity analysis by sequentially excluding one study at a time to detect the influence of a single study on the overall estimate. The results displayed that no study disproportionately affected the summary risk estimates in this meta-analysis (Figure 3B). The eleven study-specific ORs ranged from a low of 1.72 (95%CI 1.11–2.68) to 2.27 (95%CI 1.58–3.27) via the omission of the study by Pekmezovic et al. [8] and the study by Brown et al. [17], respectively.

### Cumulative meta-analysis

**Figure 4:**
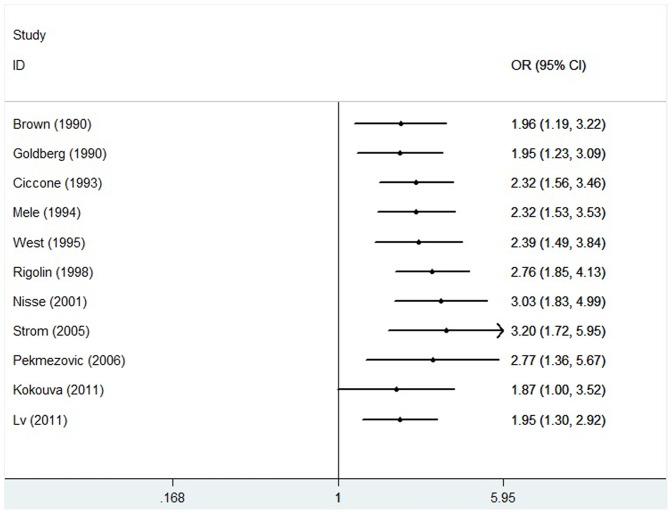
Forest plots showing the result of the cumulative meta-analysis.

Cumulative meta-analysis of the relationship between pesticide exposure and risk of MDS was also implemented by sorting the studies based on publication time. Figure 4 showed the results from the cumulative meta-analysis of this connection in chronologic order. The 95% CIs became increasingly narrower with each addition of more data, suggesting the precision of each estimate was progressively increasing with the addition of more cases.

### Publication bias

As reflected by the funnel plot (Begg’s test, *P* = 0.350) and the Egger’s test (*P* = 0.113), there was no publication bias being discovered. The data witnessed our result was statistically robust.

## Discussion

The first myelodysplastic syndromes (MDS) case series was reported about 40 years ago [26]. Thus, the recognition of MDS is approximately 100 years behind the recognition of other hematologic malignancies. Similarly, the level of epidemiologic knowledge of MDS is far below that of other cancers. Therefore, further investigation of risk factors in MDS patients is needed to improve MDS prevention.

Pesticides are widely applied in agriculture all over the world. Three million cases of acute severe pesticide poisoning and over 200,000 deaths are reported annually [4]. Pesticides are thus considered a risk factor for some cancers. Moreover, two previous meta-analyses have been performed in hematological malignancies, which indicated that pesticide exposure could increase risk of non-Hodgkin lymphoma, leukemia and multiple myeloma [27], [28]. However, the result about MDS and pesticide exposure was limited. Recent epidemiological studies have examined the potential association between pesticide exposure and the risk of MDS, but none of the results has been conclusive. We attempted to clarify this possible relationship through a meta-analysis of eleven case-control studies.

To the best of our knowledge, this is the first meta-analysis assessing the relationship between pesticide exposure and MDS. Several interesting points raised by our analysis are worth discussing. Firstly, our research demonstrated a significantly positive correlation between pesticide exposure and MDS, which indicated pesticide exposure was associated with a 95% increased risk of MDS. Sensitivity analysis and cumulative analysis confirmed the robustness of our outcomes. In addition, subgroup analyses showed a stronger effect of pesticide exposure on RA/RARS than on RAEB/RAEBt (i.e., exposed MDS patients had 63% increased risk of RA/RARS and 49% increased risk of RAEB/RAEBt, respectively). Our study also illustrated that exposure to insecticides can the increase risk of MDS by 71%, while exposure to herbicides (OR = 1.16, 95%CI 0.55–2.43) and fungicides (OR = 0.70, 95%CI 0.20–3.20), respectively, add no risk. Our subset analysis according to geographical region noted higher risk of MDS in Europe (113%) than in Asia (100%) and the United States (52%).

The biological mechanism underlying the linkage of pesticide exposure to the pathogenesis of MDS remains largely unknown. However, several mechanisms are conceivable. Exposure to pesticides might cause overexpression of reactive oxygen species (ROS) sufficient to overwhelm antioxidant defense mechanisms and thereby lead to extensive DNA damage, protein damage, and hematopoietic irregularities [29]. On the other hand, pesticides might bind to and displace endogenous ligands of steroid nuclear receptors, including estrogen and androgen receptors, thus aberrantly activating receptor function and inducing changes in gene expression networks [30]. Recent in vitro mechanistic studies offer novel insight. For example, Boros and Williams reported that exposure of leukemic cell lines (K562) to increasing doses of an organophosphate pesticide (isofenphos) resulted in dose-dependent leukemic cell proliferation [31]. In addition, some previous studies demonstrating that pesticide exposure could induce chromosomal defects [32], [33], might also suggest that pesticides could increase the risk of developing MDS. Further research is warranted to elucidate the likely biological mechanisms.

As a meta-analysis of previously published observational studies, our research has some limitations that influence interpretation of the results. First, although the present results seemed to suggest the absence of publication bias, our meta-analysis was vulnerable to publication bias, because only studies published in English were included. Limited resources prevented us from including articles published in other languages and databases. Second, no prospective studies of the association between pesticide exposure and MDS risk were available, and all included studies had a retrospective case-control design. Thus, owing to the limitations of case-control design, the possibility of undetected bias could not be excluded. Third, it is known farmers in China use large amounts of pesticides and this is an ideal population to study their effect on health. However, only one study from China was included in this meta-analysis. Fourth, too few original studies have separated biocides into insecticides, herbicides or fungicides to justify concluding the potential existence of a relationship between exposure to one or several categories of biocides and MDS. Significantly increased risks were observed, with an apparently higher increase when the exposure was to insecticides.

In summary, our findings support that pesticide exposure is associated with the increased risk of MDS, and this association varies widely across disease subtype, geographic region and specific biocide category. Larger and more rigorous analytical studies will be warranted to generate more robust conclusion to guide clinical practice in MDS prevention in the future.

## Supporting Information

Checklist S1 **PRISMA checklist.**
(DOC)
